# The role of macrophage migration inhibitory factor in promoting benign prostatic hyperplasia epithelial cell growth by modulating COX-2 and P53 signaling

**DOI:** 10.1242/bio.053447

**Published:** 2020-11-12

**Authors:** Hualin Song, Qi Shen, Shuai Hu, Jie Jin

**Affiliations:** 1 Department of Urology, Peking University First Hospital and Institute of Urology, Peking University, 100034 Beijing, China; 2 National Research Center for Genitourinary Oncology, 100034 Beijing, China; 3 Beijing Key Laboratory of Urogenital Diseases (male), Molecular Diagnosis and Treatment Center, 100034 Beijing, China

**Keywords:** Benign prostatic hyperplasia, Proliferation, MIF, COX-2, P53

## Abstract

Inflammation and proinflammatory cytokines have been implicated in the progression of benign prostatic hyperplasia (BPH). Macrophage migration inhibitory factor (MIF) is a proinflammatory cytokine. Our previous study found that MIF is highly expressed in BPH epithelium. It has been reported that there is a correlation between MIF and clinical BPH progression. However, whether MIF has an effect on BPH epithelial cells is not clear. The aim of this study was to explore whether MIF has a role in BPH. Our results showed that immunohistochemistry (IHC) showed that MIF is highly expressed in the epithelium and that MIF and PCNA expression levels are higher in BPH samples than in control. CCK8 and flow cytometry assays showed that recombinant human MIF (rMIF) promoted the proliferation of BPH-1 and PWR-1E cells, while ISO-1 partially reversed this effect on proliferation. JC-1 assays showed that rMIF inhibited the apoptosis of BPH-1 and PWR-1E cells, and ISO-1 could partially reverse this inhibition. Moreover, western blotting indicated that rMIF downregulated P53 and upregulated COX-2. Furthermore, MIF-induced proliferation could be inhibited by celecoxib in the CCK8 and flow cytometry assay. MIF-inhibited apoptosis could be partially reversed by celecoxib in the JC-1 assay. Western blotting showed that celecoxib could partially reverse MIF-induced COX-2 upregulation and P53 downregulation. Together, MIF is highly expressed in BPH epithelium. *In vitro*, MIF promoted BPH epithelial cell growth by regulating COX-2 and P53 signaling. Targeting MIF may provide a new option for the improved treatment of BPH in the future.

## Abstract (precis)

**Summary:** This study found high levels of MIF expression in BPH epithelium. MIF promoted BPH epithelial cell growth and participated in the progression of BPH by regulating COX-2 and P53 signaling

## INTRODUCTION

Benign prostatic hyperplasia (BPH) is the most common disease in older men (Wei et al., 2008). BPH occurs in 15% to 60% of men over the age of 40 years and in more than 70% of men older than 70 years (Parsons et al., 2008; Welliver et al., 2020). It usually occurs with lower urinary tract symptoms (LUTS) and seriously affects the quality of life of patients due to dysuria, nocturia, urinary retention and other symptoms (Coyne et al., 2009; Wei et al., 2008; Welliver et al., 2020). The annual health care costs associated with this disease are high, posing a heavy financial burden on the patient's family and society. As the global population ages, BPH will increasingly become a very important public health problem worldwide. However, the pathogenesis of BPH is still largely unclear. Inflammation and proinflammatory cytokines are recognized to be associated with the growth of the prostate (Nickel et al., 2008; Robert et al., 2009).

Macrophage migration inhibitory factor (MIF) was originally identified as a product isolated from activated T lymphocyte culture supernatants and was characterized as a cytokine that inhibited the random migration of macrophages (Bloom and Bennett, 1966). Activated T cells are considered to be the source of MIF, and monocyte/macrophage populations are the targets of its inhibitory effects against migration (Mitchell et al., 2002). It has been reported that MIF could promote the proliferation of prostate cancer and play an important role in the development of other diseases (Fu et al., 2019; Hussain et al., 2013; Kong et al., 2018; Tawadros et al., 2013). Meyer-Siegler et al. reported MIF expression in the epithelium of the prostate (Meyer-Siegler et al., 1998). We also found that MIF is highly expressed in epithelial cells in BPH. Latil et al. carried out an international, randomized, double-blind, parallel-group, tamsulosin-controlled study in 206 men with BPH-associated LUTS and showed that anti-inflammatory treatment by Permixon could reduce MIF expression and the international prostate symptom score (IPSS) in BPH patients (Latil et al., 2015). Moreover, in this study, patients with MIF overexpression at baseline were more responsive to Permixon as evidenced by IPSS than those who did not overexpress MIF. However, whether MIF has an effect on the epithelial cells in BPH and its possible mechanism are not clear. Therefore, in this study, we aimed to explore the role of MIF in BPH epithelial cells.

## RESULTS

### Immunolocalization and expression of MIF and proliferating cell nuclear antigen (PCNA) in BPH samples and control

**Fig. 1.**
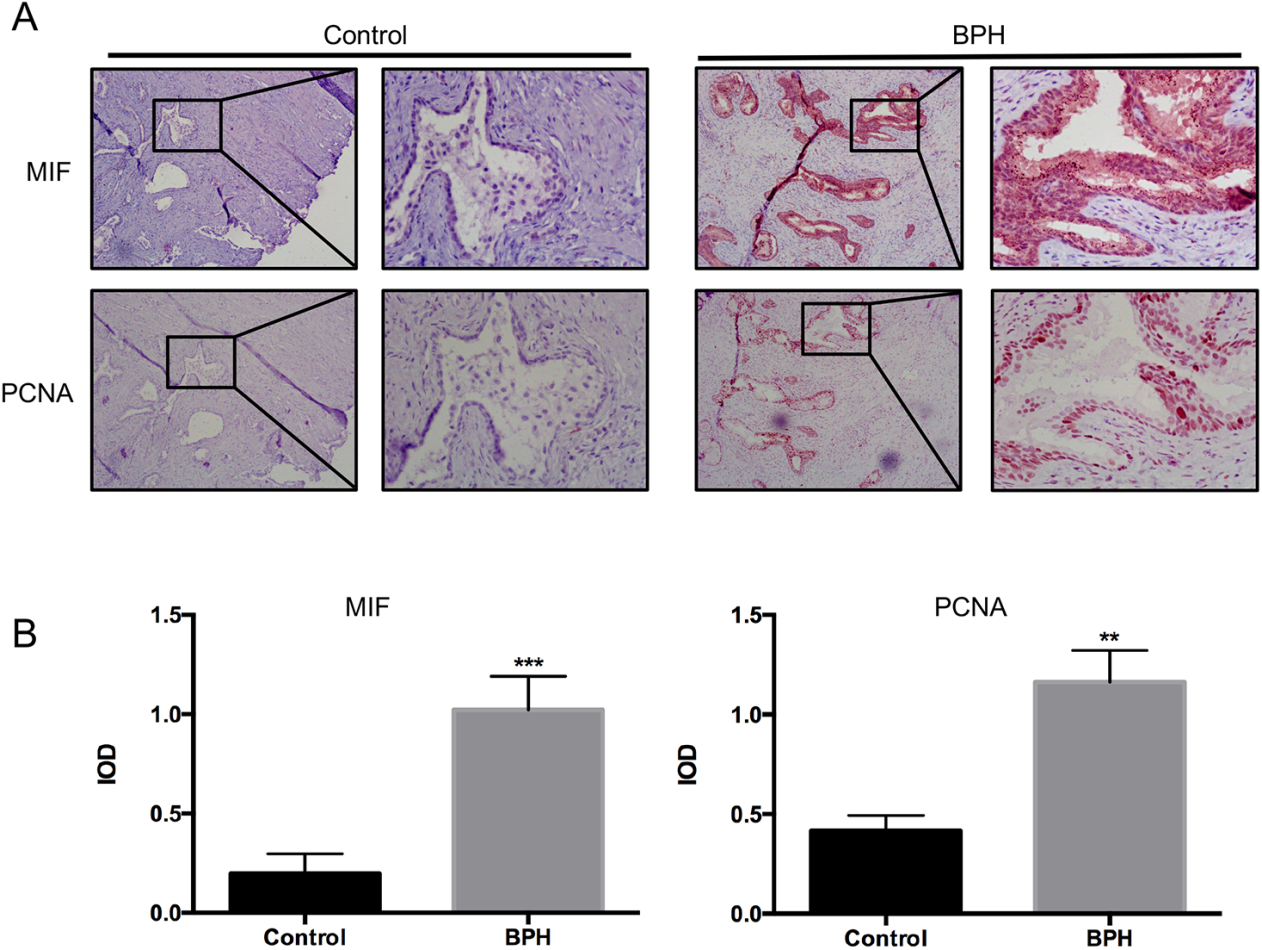
**MIF and PCNA expression in BPH samples and control.** (A) Representative IHC images of MIF and PCNA expression in BPH samples and control. Left: 100×; right: 400×. (B) Histogram shows MIF and PCNA expression in BPH samples and control. ***P<0.001, **P<0.01. *P*-value was calculated using two-tailed Student's *t*-test.

Thirty patients were selected stochastically from BPH patients who underwent transurethral resection of the prostate (TURP) between January 2010 and December 2017 at Peking University First Hospital. Immunohistochemistry (IHC) was used to measure MIF and proliferation marker PCNA expression in BPH samples and control. IHC showed that MIF was highly expressed in the epithelium and MIF expression was higher in BPH than control (Fig. 1A). Quantification of MIF expression showed that MIF was significantly higher in BPH samples than control (*P*<0.001, Fig. 1B). IHC showed that PCNA expression were higher in BPH samples and control (Fig. 1A). Quantification of PCNA expression showed that PCNA was significantly higher in BPH samples than control (*P*<0.01, Fig. 1B). In addition, IHC showed MIF and PCNA were localized to the same areas (Fig. 1A). These results indicated that MIF might be related to the proliferation of BPH epithelial cells.

### MIF promoted the growth of BPH epithelial cells

**Fig. 2.**
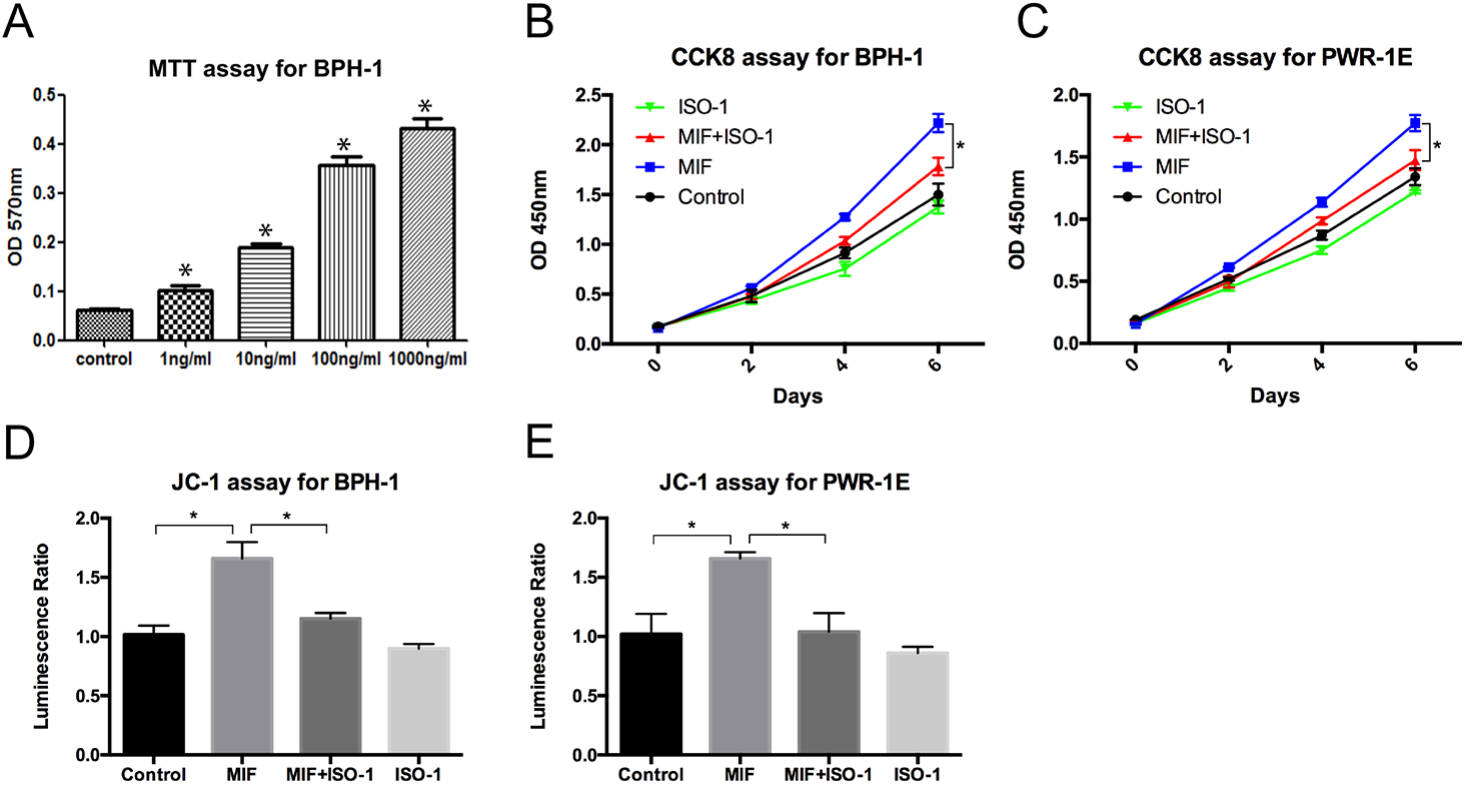
**MIF promoted proliferation of BPH-1 and PWR-1E cells.** (A) MTT assay showed the proliferation of BPH-1 cells treated with various concentrations of rMIF for 3 days. Data are presented as mean±s.d., *n*=3. (B) CCK8 assay showed the proliferation of BPH-1 cells treated with control, rMIF (100 ng/ml), rMIF (100 ng/ml)+ISO-1 (10 µM) and ISO-1 (10 µM), respectively. Data are presented as mean±s.d., *n*=3. (C) CCK8 assay showed the proliferation of PWR-1E cells treated with control, rMIF, rMIF+ISO-1 and ISO-1, respectively. Data are presented as mean±s.d., *n*=3. (D) JC-1 assay showed the growth rates of the BPH-1 cells treated by control, rMIF, rMIF+ISO-1 and ISO-1, respectively. Data are presented as mean±s.d., *n*=3. (E) JC-1 assay showed the growth rates of the PWR-1E cells treated by control, rMIF, rMIF+ISO-1 and ISO-1, respectively. Data are presented as mean±s.d., *n*=3. *P<0.05, *P<0.05. Statistical analyses were performed using ANOVA followed by Tukey's test for multiple comparison.

**Fig. 3.**
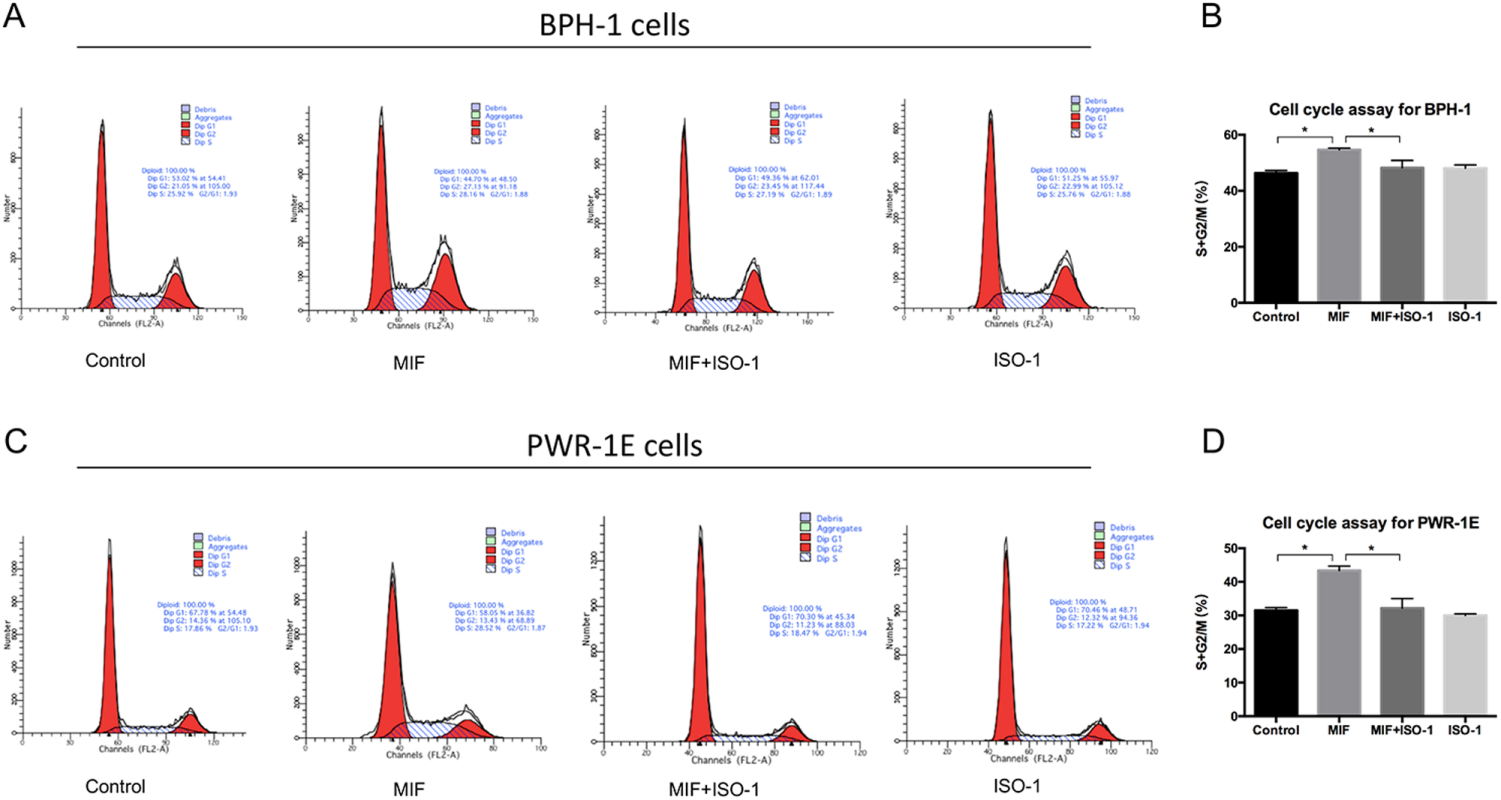
**Flow cytometry test that MIF promoted proliferation of BPH-1 and PWR-1E cells.** (A) Flow cytometry showed cell cycle in BPH-1 cells treated by control, rMIF, rMIF+ISO-1 and ISO-1, respectively. (B) Histogram for S+G2/M phase of the cell cycle results in BPH-1 cells. Data are presented as mean±s.d., *n*=3. (C) Flow cytometry showed cell cycle in PWR-1E cells treated by control, rMIF, rMIF+ISO-1 and ISO-1, respectively. (D) Histogram for S+G2/M phase of the cell cycle results in PWR-1E cells. Data are presented as mean±s.d., *n*=3. *P<0.05. Statistical analyses were performed using ANOVA followed by Tukey's test for multiple comparison.

To study the effect of MIF on the proliferation of BPH epithelial cells *in vitro*, recombinant human MIF (rMIF) and MIF inhibitor ISO-1 were used to treat BPH-1 and PWR-1E cells. The MTT assay was used to measure the growth rates of BPH-1 cells treated with different concentrations of rMIF for 3 days, and the results showed that the higher the rMIF concentration was, the higher the growth rate of BPH-1 cells (Fig. 2A). Then, we set up the following four groups: control, rMIF (100 ng/ml), rMIF (100 ng/ml)+ISO-1 (10 µM) and ISO-1 (10 µM). The CCK8 assay showed that rMIF treatment significantly upregulated the growth rates of the BPH-1 and PWR-1E cells, while rMIF+ISO-1 treatment partially reversed the growth rates (Fig. 2B,C). JC-1 assay monitors mitochondria health. Mitochondrial disruption is an early sign of apoptosis. The JC-1 assay showed that rMIF significantly inhibited the apoptosis of BPH-1 and PWR-1E cells, and rMIF+ISO-1 partially reversed this inhibition (Fig. 2D,F). The cell cycle assay with flow cytometry showed the percentages of cells in the G1, G2 and S phases in the four groups. In cell cycle assay, the increase in S-phase and G2/M phase is correlated with proliferation. Cells treated with rMIF showed a significant increase in S phase and G2/M phase DNA content, but treated with rMIF+ISO-1 partially reversed the increase in BPH-1 (Fig. 3A,B; Fig. S1) and PWR-1E cells (Fig. 3C,D). Taken together, these results suggested that MIF could promote the growth of BPH-1 cells *in vitro*.

### MIF modulated the COX-2 and P53 signaling pathways

**Fig. 4.**
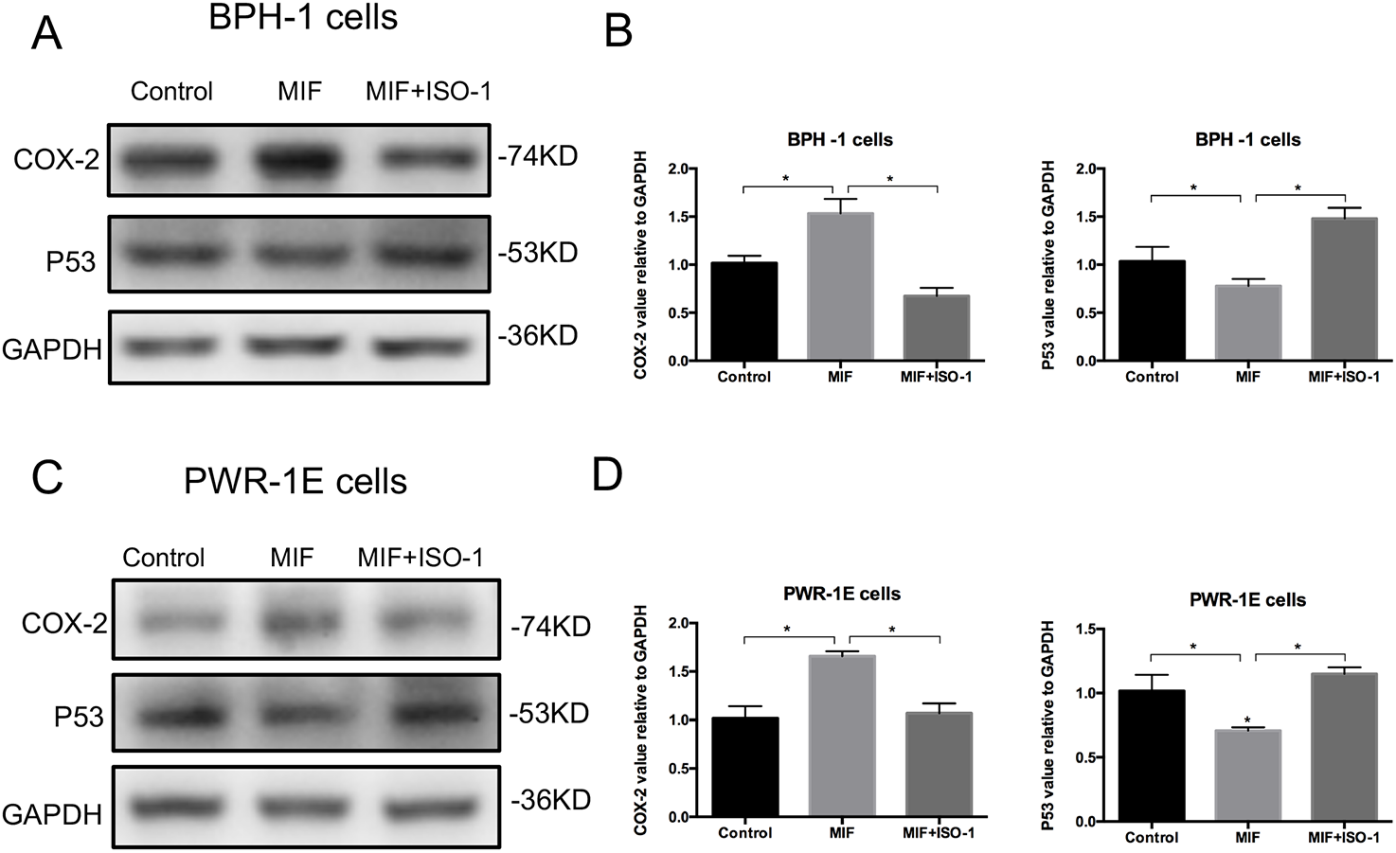
**MIF modulation COX-2 and P53 signaling pathway.** (A) Western blotting detected the expression of COX-2 and P53 in BPH-1 cells treated by control, rMIF and rMIF+ISO-1, respectively. (B) Western blot analysis: the expression of COX-2 and P53 in BPH-1 cells treated by control, rMIF and rMIF+ISO-1. Data are presented as mean±s.d., *n*=3. (C) Western blotting detected the expression of COX-2 and P53 in PWR-1E cells treated by control, rMIF and rMIF+ISO-1, respectively. (D) Western blot analysis: the expression of COX-2 and P53 in PWR-1E cells treated by control, rMIF and rMIF+ISO-1. Data are presented as mean±s.d., *n*=3. *P<0.05. Statistical analyses were performed using ANOVA followed by Tukey's test for multiple comparison.

**Fig. 5.**
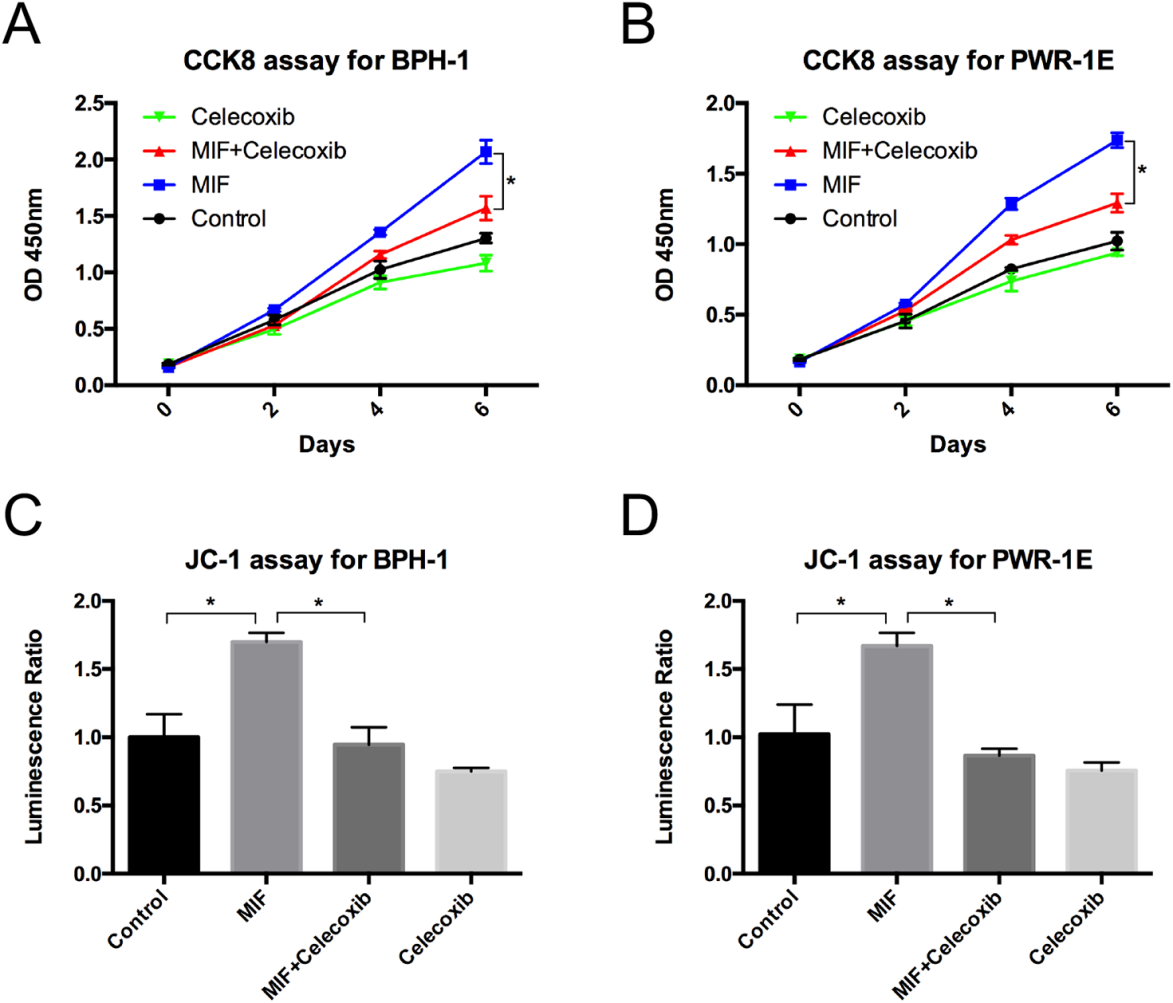
**COX-2 is a key factor of MIF promoted proliferation.** (A) CCK8 assay showed the proliferation of BPH-1 cells treated with control, rMIF (100 ng/ml), rMIF (100 ng/ml)+celecoxib (5 µM) and celecoxib (5 µM), respectively. Data are presented as mean±s.d., *n*=3. (B) CCK8 assay showed the proliferation of PWR-1E cells treated with control, rMIF, rMIF+celecoxib and celecoxib, respectively. Data are presented as mean±s.d., *n*=3. (C) JC-1 assay showed the growth rates of the BPH-1 cells treated by control, rMIF, rMIF+celecoxib and celecoxib, respectively. Data are presented as mean±s.d., *n*=3. (D) JC-1 assay showed the growth rates of the PWR-1E cells treated by control, rMIF, rMIF+celecoxib and celecoxib, respectively. Data are presented as mean±s.d., *n*=3. *P<0.05. Statistical analyses were performed using ANOVA followed by Tukey's test for multiple comparison.

**Fig. 6.**
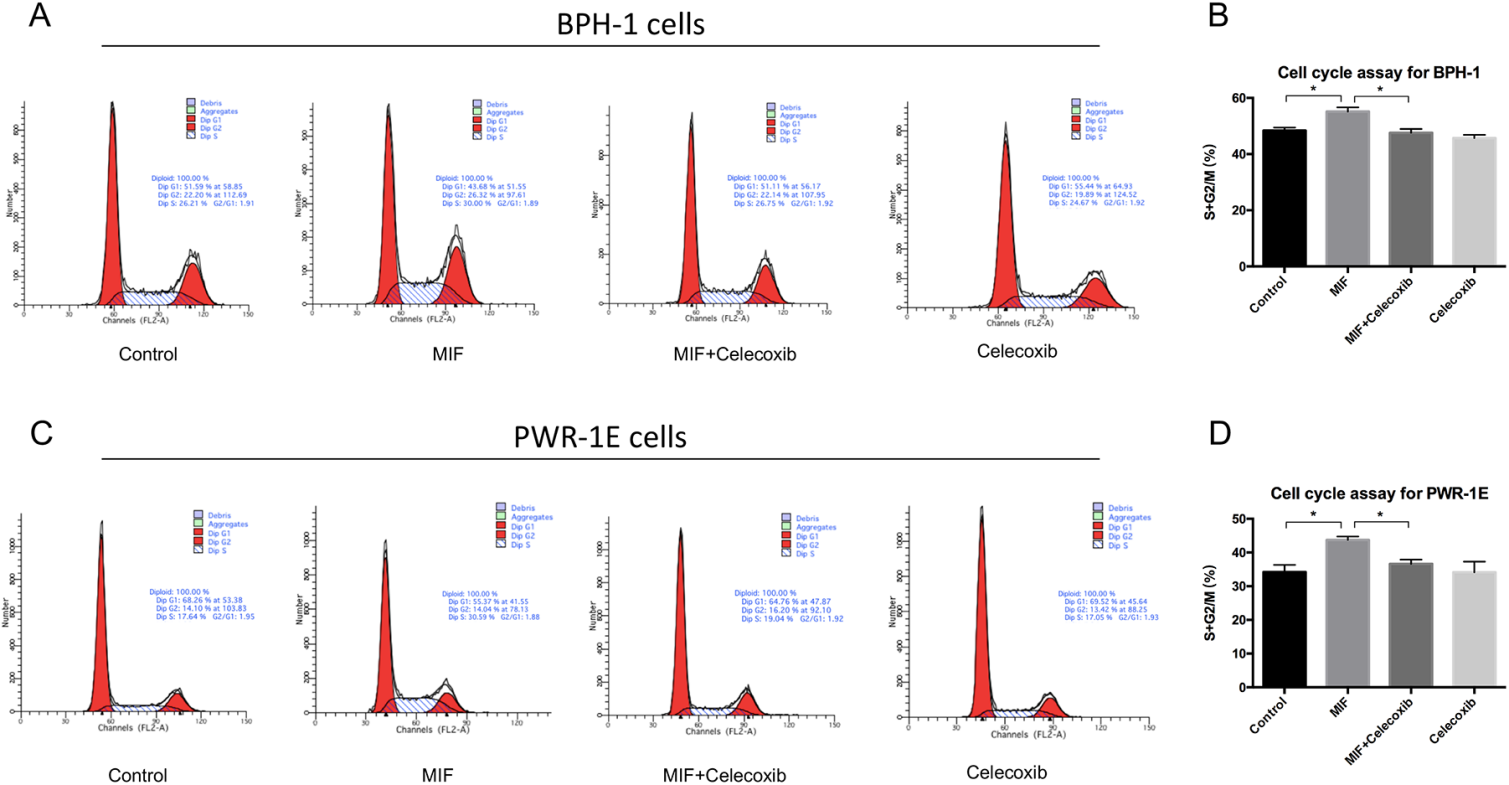
**Flow cytometry test that COX-2 is a key factor of MIF promoted proliferation.** (A) Flow cytometry showed cell cycle in BPH-1 cells treated by control, rMIF, rMIF+celecoxib and celecoxib, respectively. (B) Histogram for S+G2/M phase of the cell cycle results in BPH-1 cells. Data are presented as mean±s.d., *n*=3. (C) Flow cytometry showed cell cycle in PWR-1E cells treated by control, rMIF, rMIF+celecoxib and celecoxib, respectively. (D) Histogram for S+G2/M phase of the cell cycle results in PWR-1E cells. Data are presented as mean±s.d., *n*=3. *P<0.05. Statistical analyses were performed using ANOVA followed by Tukey's test for multiple comparison.

**Fig. 7.**
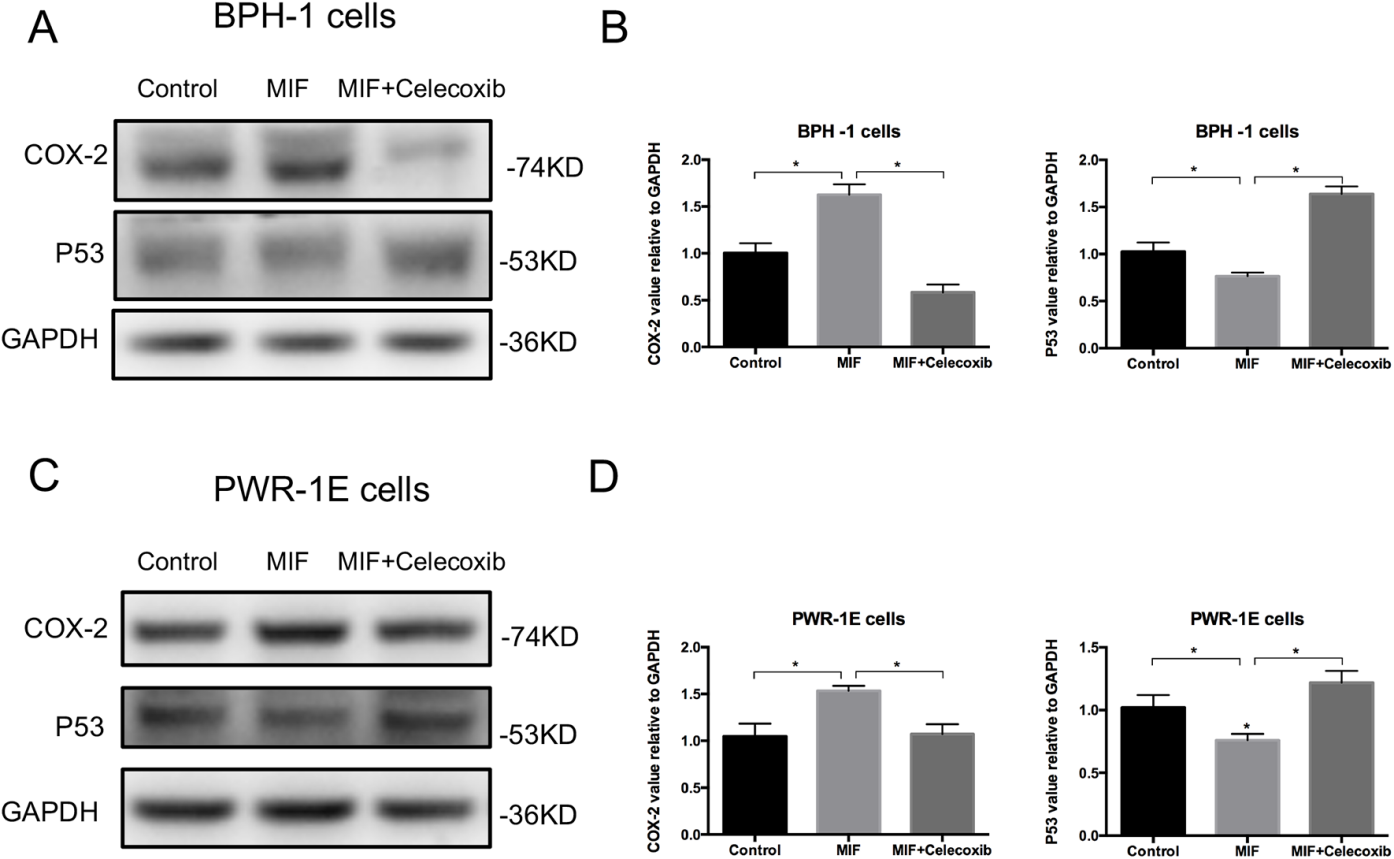
**Expression of signaling molecules COX-2 and P53 involved in the inhibition of COX-2.** (A) Western blotting detected the expression of COX-2 and P53 in BPH-1 cells treated by control, rMIF and rMIF+celecoxib, respectively. (B) Western blot analysis: the expression of COX-2 and P53 in BPH-1 cells treated by control, rMIF and rMIF+celecoxib. Data are presented as mean±s.d., *n*=3. (C) Western blotting detected the expression of COX-2 and P53 in PWR-1E cells treated by control, rMIF and rMIF+celecoxib, respectively. (D) Western blot analysis: the expression of COX-2 and P53 in PWR-1E cells treated by control, rMIF and rMIF+celecoxib. Data are presented as mean±s.d., *n*=3. *P<0.05. Statistical analyses were performed using ANOVA followed by Tukey's test for multiple comparison.

**Fig. 8.**
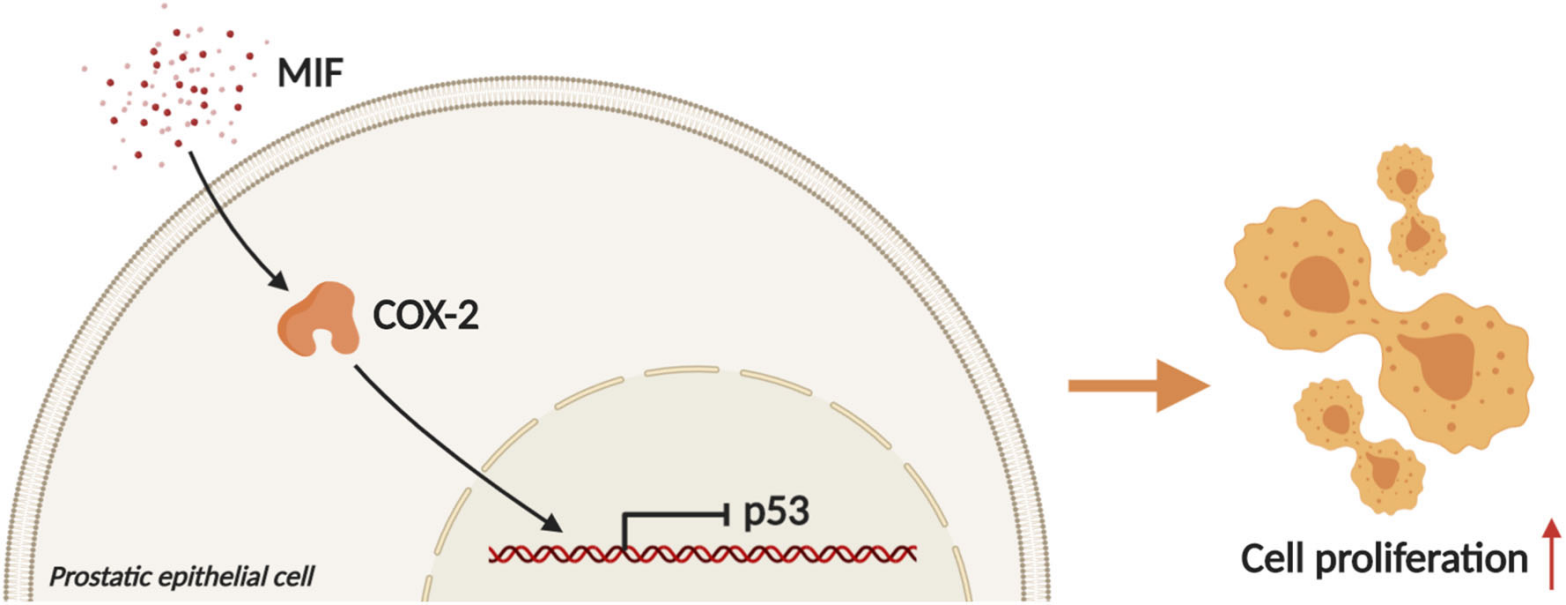
**Mechanism and regulatory pathway of MIF promoting BPH epithelial cells growth.** MIF promotes the growth of BPH epithelial cells by downregulating P53 via upregulating COX-2. (Created with BioRender.com).

Western blotting analysis showed that the expression of COX-2 was significantly upregulated and the expression of P53 was downregulated in BPH-1 and PWR-1E cells with rMIF treatment, while in BPH-1 and PWR-1E cells treating with rMIF+ISO-1, the changed expression of COX-2 and P53 were partially reversed (Fig. 4A–D). The CCK8 assay showed that rMIF treatment significantly upregulated proliferation, while rMIF+COX-2 inhibitor celecoxib (5 µM) treatment partially reversed the proliferation in BPH-1 (Fig. 5A) and PWR-1E cells (Fig. 5B). The JC-1 assay showed that rMIF+celecoxib partially reversed the inhibition of rMIF induced apoptosis in BPH-1 (Fig. 5C) and PWR-1E cells (Fig. 5D). The cell cycle assay showed that rMIF+celecoxib partially reversed the rMIF induced increase in S phase and G2/M phase DNA content in BPH-1 (Fig. 6A,B; Fig. S2) and PWR-1E cells (Fig. 6C,D). Furthermore, western blotting analysis showed that in rMIF treatment, the expression of COX-2 was significantly upregulated and the expression of P53 was downregulated compared with control, while in rMIF+celecoxib treatment, the expression of COX-2 and the expression of P53 were partially reversed in BPH-1 (Fig. 7A,B) and PWR-1E cells (Fig. 7C,D). These lines of evidence indicated that COX-2 and P53 might play an important role in the MIF-mediated promotion of BPH-1 cell proliferation (Fig. 8).

## DISCUSSION

Through the above experiments, we found that MIF may regulate COX-2/P53 to promote the growth of BPH epithelial cells and accelerate the progression of BPH. MIF is a proinflammatory cytokine that recently emerged (Mitchell et al., 2002). Some studies have shown that MIF can promote tumor growth and other diseases (Bach et al., 2008; Grieb et al., 2010; Richard et al., 2015). It has been reported that there is a correlation between MIF and clinical BPH progression (Latil et al., 2015; Meyer-Siegler et al., 1998). However, it is not clear whether MIF has an effect on BPH. In the present study, we identified that the expression of MIF was higher in BPH tissue samples than in control. IHC revealed that MIF was associated with the proliferation marker PCNA. These results indicated that MIF might be related to the proliferation of BPH epithelium.

We further explored the role of MIF *in vitro* and found that MIF could promote BPH epithelial cell growth. It has been reported that MIF could promote cell growth in some diseases, such as cervical adenocarcinoma or neural stem/progenitor cells (Ohta et al., 2012, 2016; Zhang et al., 2012). Here, we found that MIF could promote the proliferation of the BPH-1 cell line. Therefore, MIF might play a certain role in the development of BPH.

We further investigated the molecular mechanism by which MIF promoted BPH cell proliferation. We found that MIF promoted BPH proliferation by modulating COX-2 and P53 signaling. It has been reported that MIF can affect tumor progression through some mechanisms, including interactions with COX-2 and P53 (Bach et al., 2008; Mitchell et al., 2002; Rizzo, 2011; Vousden and Prives, 2009; Xia et al., 2005). Regarding COX-2, MIF is implicated in an increase in the expression of the gene encoding COX-2 (de Dios Rosado and Rodriguez-Sosa, 2011). Mawhinney et al. found that MIF led to a significant increase in COX-2 production (Mawhinney et al., 2015). In the present study, we found that MIF was associated with COX-2 in clinical samples and that MIF could regulate COX-2 *in vitro*. Considering the interaction of MIF and COX-2, we investigated the role of P53 in this study. Mitchell et al. reported that MIF inhibited P53 activity in monocytes, in a process involving COX-2 (Mitchell et al., 2002). Han et al. showed that P53-induced apoptosis and activity were enhanced in COX-2 null cells but not in wild-type cells (Han et al., 2002). Subbaramaiah et al. reported that P53 inhibited the expression of COX-2, while a mutation in TP53 resulted in an increase in COX-2 activity (Subbaramaiah et al., 1999). Wu et al. found that COX-2 inhibits apoptosis via P53 signaling (Wu et al., 2016). In this study, we found that MIF was associated with P53 in clinical samples. We further verified the association between MIF and P53 and the relationship between COX-2 and P53 *in vitro*. These results indicated that MIF may promote proliferation in BPH by modulating COX-2 and P53 signaling.

This study has several limitations. The cells used in this study are BPH-1 cells and PWR-1E cells. These cell lines were immortalized by large T antigen (SV40). This immortalization inhibits tumor suppressor genes and activates the carcinogenic pathway. The cell lines described retain epithelial characteristics and are tumorigenic but nonmetastatic. Therefore, they cannot fully represent the biological characteristics of epithelial cells in BPH (Hayward et al., 2001). However, these two cell lines are also commonly used cell lines in the field of BPH research.

### Conclusion

In summary, this study found high levels of MIF expression in BPH epithelium. *In vitro*, MIF promoted BPH epithelial cell growth and participated in the progression of BPH by regulating COX-2 and P53 signaling. Anti-inflammation therapies via targeting the MIF in patients with BPH may be warranted in the future.

## MATERIALS AND METHODS

### Cell culture

PWR-1E cell line was purchased from the American Type Culture Collection (ATCC, USA). The cells were cultured in complete keratinocyte serum-free medium (Invitrogen, USA) was used. The BPH-1 cell line was purchased from Keygen Biotech (KG1008, China). The cells were maintained in RPMI-1640 medium (01-100-1ACS, Biological Industries, Israel) containing 1% streptomycin and 1% penicillin and supplemented with 10% fetal bovine serum (04-001-1ACS, Biological Industries, Israel). The two cell lines were cultured in a humidified incubator at 37°C with 5% carbon dioxide.

### Patients

Thirty BPH patients were stochastically selected from the electronic medical record system of IHC. These patients underwent TURP between January 2010 and December 2016 at Peking University First Hospital. This study excluded patients with urinary tract infections, a history of urethral catheterization, previous prostate-related surgery, prostatitis or prostate cancer. Prostate tissues were microscopically examined by two independent pathologists to finalize the diagnosis of BPH. Age-matched prostate tissues, acquired from patients with bladder cancer who underwent radical cystectomy and prostatectomy have been added as control with no BPH to compare to those with BPH. The use of human samples in this study was approved by the Ethics Committee of Peking University First Hospital. The research involving human participants experiments had been approved by our hospital and our equivalent committee. The participants provided their written informed consents to participate in this study.

### IHC

The expression levels of MIF and PCNA were analyzed by IHC of continuous paraffin sections to study the relationship between MIF and the proliferative state of prostate epithelial cells. Prostate tissues were microscopically examined by two independent urologic pathologists to finalize the division. The TURP-operated prostate tissue was fixed in 4% formalin buffer at 4°C overnight, dehydrated, and conventionally embedded in paraffin. Then the sections were cut into 5 μm sections. After dewaxing and hydration, 3% hydrogen peroxide was used to inactivate endogenous peroxidase. The primary antibodies MIF (sc-271631, Santa Cruz Biotechnology, USA; 1:500 dilution) and PCNA (#13110, Cell Signaling Technology, USA; 1:2000 dilution) were incubated overnight at 4°C. The primary antibody was recognized by a biotinylated secondary antibody for 30 min at room temperature and visualized by a Vectastain ABC system and a peroxidase substrate DAB kit. The mean immune response scores of MIF and PCNA (IOD) were analyzed by using Image-Pro Plus 6.0 software (Media Cybernetics, Rockville, MD, USA).

### MTT assay

Six thousand BPH-1 or PWR-1E cells were seeded into one well of a 24-well plate. The cells were collected on day 3. To perform the MTT assay, 100 µl of 5 mg/ml MTT was added to one well. The cells were cultured for 4 h in a 37°C incubator. The medium was removed, and then 150 µl of DMSO was added. The plate was covered with tin foil. Then, the plate was oscillated on an orbital oscillator for 15 min. The absorbance was measured at 570 nm.

### CCK8 assay

BPH-1 cells and PWR-1E cells were plated on 96-well plates (1000 cells per well) overnight and rMIF (300-69, PeproTech, USA; 100 ng/ml), MIF inhibitor ISO-1 (S7732, Selleck, USA; 10 µM), COX-2 inhibitor celecoxib (S1261, Selleck, USA; 5 µM) or nothing was added into each well. BPH-1 and PWR-1E cell growth was assessed on days 2, 4, and 6 using the Cell Counting Kit-8 (CCK8) (CK04; Dojindo Molecular Technologies, Tokyo, Japan) according to the manufacturer's protocol. The absorbance value was measured using a Varioskan Flash plate reader (Thermo Fisher Scientific, Waltham, MA, USA) at 450 nm.

### JC-1 assay

Fifteen thousand BPH-1 and PWR-1E cells were plated in each well of 96-well plates, and rMIF (300-69, PeproTech, USA; 100 ng/ml), ISO-1 (S7732, Selleck, USA; 10 µM), celecoxib (S1261, Selleck, USA; 5 µM) or nothing was added into each well. One day later, 10 µg/ml JC-1 solution was added to each well. The cells were then incubated for 20 min in a 37°C incubator. We detected JC-1 using a fluorescence microscope. To visualize the JC-1 monomer, the excitation light was set to 488 nm, and the emission light was set to 530 nm. To visualize the JC-1 polymer, the excitation light was set to 529 nm, and the emission light was set to 590 nm. In the end, JC-1 assay was used to obverse the situation of cell apoptosis.

### Flow cytometry

One hundred thousand BPH-1 and PWR-1E cells were seeded into each well of a six-well plate, and rMIF (300-69, PeproTech, USA; 100 ng/ml), ISO-1 (S7732, Selleck, USA; 10 µM), celecoxib (S1261, Selleck, USA; 5 µM) or nothing was added into each well. The cells were collected on the second day. Then, the cells were added to 5 ml of 70% (precooled) ethanol and stored at 4°C to fix overnight. The fixed cells were collected, and RNAse (10 mg/ml in 1 mol/l Trisc1, pH 7.4) was added. The cells were then incubated for 30 min at 37°C in water. Propidium Iodide (PI) (450 μl PI per sample undiluted) was added to the cells and then incubated for 30 min on ice. Then, flow cytometry was used to analyze the cells, Cellquest software was used to obtain data, and Modfit software was used to analyze the cell cycle.

### Western blotting assay

The cells were lysed with RIPA buffer containing 1 mM PMSF, 0.5% phosphatase inhibitors and 1% protease inhibitors (KGP250, Keygen Biotech, China). We loaded 25 μg protein in each sample. The protein was electrotransferred onto a nitrocellulose membrane. The membrane was blocked in Tris-buffered saline plus 5% skim milk powder for 1 h. The membrane was then incubated with the primary antibodies COX-2 (#12282, Cell Signaling Technology, USA; 1:1000 dilution), P53 (#2527, Cell Signaling Technology, USA; 1:1000 dilution) and GAPDH (60004-1-Ig, Proteintech, USA; 1:2000 dilution) in TBS-T overnight at 4°C. After washing three times in TBS-T buffer, the membrane was incubated with secondary antibody (Invitrogen) for 1 h at room temperature in TBS-T. The signal was visualized using a western blotting chemiluminescence reagent (P90719, Millipore, MA, USA).

### Statistical analysis

The data are the mean±s.d. of at least three independent experiments. One-way analysis of variance (ANOVA) was performed for statistical analysis. When comparing two groups, *P*-value was calculated using two-tailed Student's *t*-test. When comparing multiple groups, statistical analyses were performed using ANOVA followed by Tukey's test for multiple comparison. We chose to use SPSS 22.0 (SPSS Inc., Chicago, IL, USA). Statistical significance was defined as *P*<0.05.

## Supplementary Material

Supplementary information
