# Supplementary material for: The role of macrophage migration inhibitory factor in promoting benign prostatic hyperplasia epithelial cell growth by modulating COX-2 and P53 signaling
Source: Biol Open. 2020 Nov 12;9(11):bio053447. doi: 10.1242/bio.053447 (PMC7673366; doi:10.1242/bio.053447)
Supplement: Supplementary information [file biolopen-9-053447-s1.pdf]

Supplemental Figure

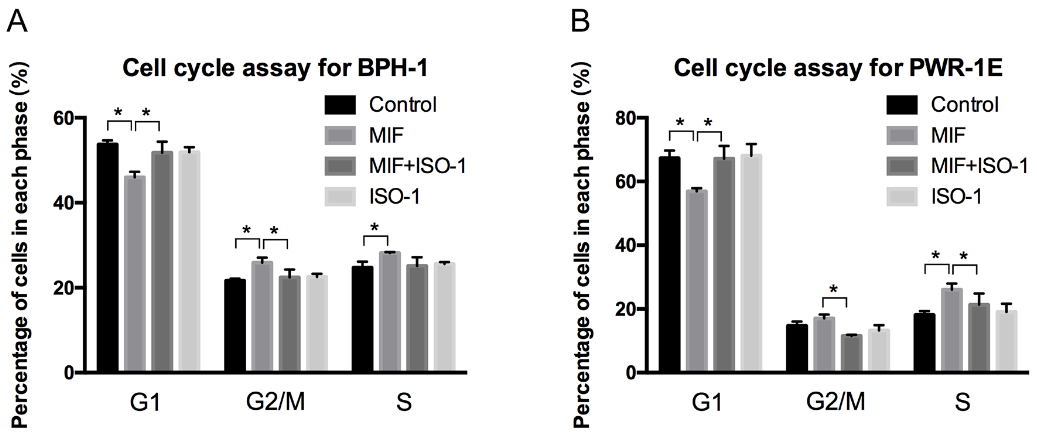

Fig. S1 Histogram of cell cycle test that MIF promoted proliferation of BPH-1 and PWR-1E cells. (A) Histogram for G1, S and G2/M phase of the cell cycle results in BPH-1 cells treated by control, rMIF, rMIF + ISO-1 and ISO-1, respectively. Data are presented as mean ± SD, n=3. (B) Histogram for G1, S and G2/M phase of the cell cycle results in PWR-1E cells treated by control, rMIF, rMIF + ISO-1 and ISO-1, respectively. Data are presented as mean ± SD, n=3. Data are presented as mean ± SD, n=3. *P< 0.05. Statistical analyses were performed using one-way analysis of variant (ANOVA) followed by Tukey's test for multiple comparison.

Biology Open • Supplementary information

Biology Open (2020): doi:10.1242/bio.053447 Supplementary information

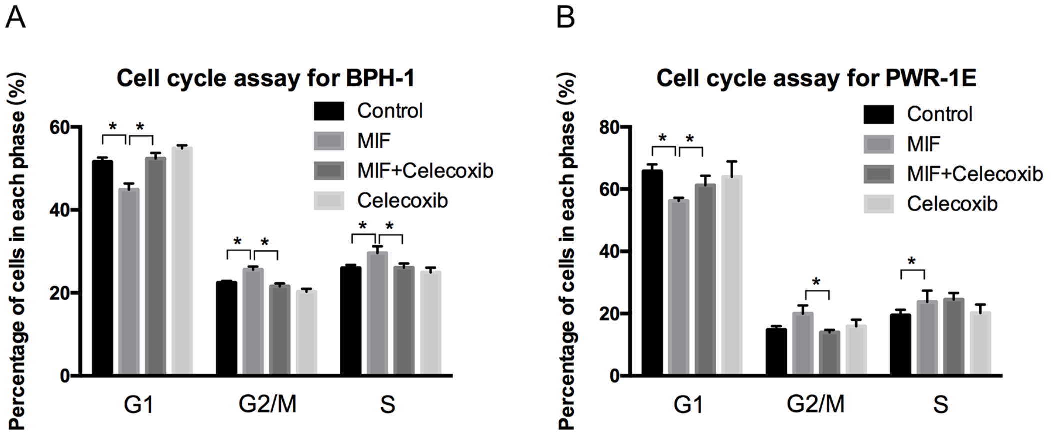

Fig. S2 Histogram of cell cycle test that COX-2 is a key factor of MIF promoted proliferation of BPH-1 and PWR-1E cells. (A) Histogram for G1, S and G2/M phase of the cell cycle results in BPH-1 cells treated with control, rMIF, rMIF + celecoxib and celecoxib, respectively. Data are presented as mean ± SD, n=3. (B) Histogram for G1, S and G2/M phase of the cell cycle results in PWR-1E cells treated with control, rMIF, rMIF + celecoxib and celecoxib, respectively. Data are presented as mean ± SD, n=3. Data are presented as mean ± SD, n=3. *P< 0.05. Statistical analyses were performed using one-way analysis of variant (ANOVA) followed by Tukey's test for multiple comparison.

Biology Open • Supplementary information
